# Women’s experience of perinatal support in a high migrant Australian population during the COVID-19 pandemic: a mixed methods study

**DOI:** 10.1186/s12884-023-05745-9

**Published:** 2023-06-09

**Authors:** Sarah J. Melov, Nelma Galas, Julie Swain, Thushari I. Alahakoon, Vincent Lee, N Wah Cheung, Therese McGee, Dharmintra Pasupathy, Justin McNab

**Affiliations:** 1 Reproduction and Perinatal Centre, Faculty of Medicine and Health, The University of Sydney, Sydney, NSW Australia; 2 Westmead Institute for Maternal and Fetal Medicine, Research and Education Building, Crn Hawkesbury and Darcy Rd, Westmead, NSW 2145 Australia; 3 Women’s and Newborn Health, Westmead Hospital, Sydney, NSW Australia; 4 Faculty of Medicine and Health, The University of Sydney, Sydney, NSW Australia; 5 Department of Renal Medicine, Westmead Hospital, Sydney, NSW Australia; 6 Westmead Applied Research Centre, Faculty of Medicine and Health, University of Sydney, Sydney, Australia; 7 Department of Diabetes & Endocrinology, Westmead Hospital, Sydney, Australia; 8 Charles Perkins Centre, The University of Sydney, Sydney, NSW Australia

**Keywords:** COVID-19, Pandemics, Maternity care, Migrants, Social Support, Antenatal Care, Gender equity, Postpartum

## Abstract

**Background:**

As a COVID-19 risk mitigation measure, Australia closed its international borders for two years with significant socioeconomic disruption including impacting approximately 30% of the Australian population who are migrants. Migrant populations during the peripartum often rely on overseas relatives visiting for social support. High quality social support is known to lead to improved health outcomes with disruption to support a recognised health risk.

**Aim:**

To explore women’s experience of peripartum social support during the COVID-19 pandemic in a high migrant population. To quantify type and frequency of support to identify characteristics of vulnerable perinatal populations for future pandemic preparedness.

**Methods:**

A mixed methods study with semi-structured interviews and a quantitative survey was conducted from October 2020 to April 2021. A thematic approach was used for analysis.

**Results:**

There were 24 participants interviewed both antenatally and postnatally (22 antenatal; 18 postnatal). Fourteen women were migrants and 10 Australian born. Main themes included; ‘Significant disruption and loss of peripartum support during the COVID-19 pandemic and ongoing impact for migrant women’; ‘Husbands/partners filling the support gap’ and *‘*Holding on by a virtual thread’. Half of the participants felt unsupported antenatally. For Australian born women, this dissipated postnatally, but migrants continued to feel unsupported. Migrant women discussed partners stepped into traditional roles and duties of absent mothers and mothers-in-law who were only available virtually.

**Conclusion:**

This study identified disrupted social support for migrant women during the pandemic, providing further evidence that the pandemic has disproportionately impacted migrant populations. However, the benefits identified in this study included high use of virtual support, which could be leveraged for improving clinical care in the present and in future pandemics. The COVID-19 pandemic impacted most women’s peripartum social support with migrant families having ongoing disruption. Gains in the pandemic included greater gender equity for domestic work as husbands/partners increased their contribution to domestic work and childcare.

**Supplementary Information:**

The online version contains supplementary material available at 10.1186/s12884-023-05745-9.

## Introduction

The COVID-19 pandemic has had a devastating impact on the world including increased direct mortality, morbidity, poverty and significant social upheaval [1, 2]. Australia experienced an initial low prevalence of COVID-19 disease compared to many other countries. The majority (89%) of COVID-19 deaths and cases in Australia during 2020 were in the state of Victoria and by mid-2021 Australia-wide there had been a total of 910 deaths [3]. Measures such as lockdowns and health facility preparedness were largely successful in containing the spread of COVID-19 until the sharp rise in cases mid-2021 [4]. Other measures for containment included closing the Australian international borders to non-citizens for nearly two years from March 20, 2020 to February 21st 2022 [5].

The closed international borders during the pandemic have impacted many aspects of life including access to overseas social support networks. This was particularly relevant for the migrant community throughout Australia. Australia has a high migrant population with 7.7 million migrants in 2020, nearly 30% of the population [6]. The main countries of birth for migrant Australians are: England (3.8%), India (2.8%), China (2.5%), New Zealand (2.2%) and the Philippines (1.2%) [6]. Of note for maternity care, the English migrant median age is 58 years while it is 35 years for Indian and 38 years for Chinese migrants [6]. Therefore, Indian and Chinese migrant women are more likely to be users of maternity services than English migrants.

Research and media reports have highlighted the impact of closed international borders on social support for migrant women in the peripartum period [7–9]. High quality social support can be a buffer against stress and consequently improve health [10]. Social support may be defined as having three main elements; emotional support from people who provide understanding and empathy; instrumental or tangible support defined as having people physically available to complete tasks for you; and people who can provide advice or knowledge, referred to as informational support [11]. Culture may also play a role in the type of, and preference for, support and how this support is provided or received [12–14].

In the Western Sydney Local Health District (WSLHD) of New South Wales (NSW) Australia, perinatal outcomes were impacted in the first year of the pandemic, including a 15% reduction in spontaneous preterm births [15]. Hospital health service changes included increased infection control measures such as mandatory mask wearing, reduced visitor access and some use of telehealth. In a low COVID-19 prevalent period, the changed obstetric outcomes are attributed largely to the indirect impact of COVID-19 through health services and societal changes. This study will assist in understanding the COVID-19 pandemic patient experience, contribute to understanding patient engagement with healthcare services and potential factors contributing to the changed perinatal outcomes during this period. Our aim was to explore the experience of peripartum support for women during the first year of the COVID-19 pandemic in a high migrant population.

## Methods

### Design

A mixed methods study design was employed with semi-structured interviews used for qualitative data collection, and quantitative data extracted from hospital pregnancy records and a study specific quantitative questionnaire. The semi-structured interview schedule focussed on three-key areas: support, cultural context and maternity care. Support during the peripartum is defined as meaning family members and others who provide emotional, tangible/instrumental (practical), and informational support [11].

### Setting

**Fig. 1:**
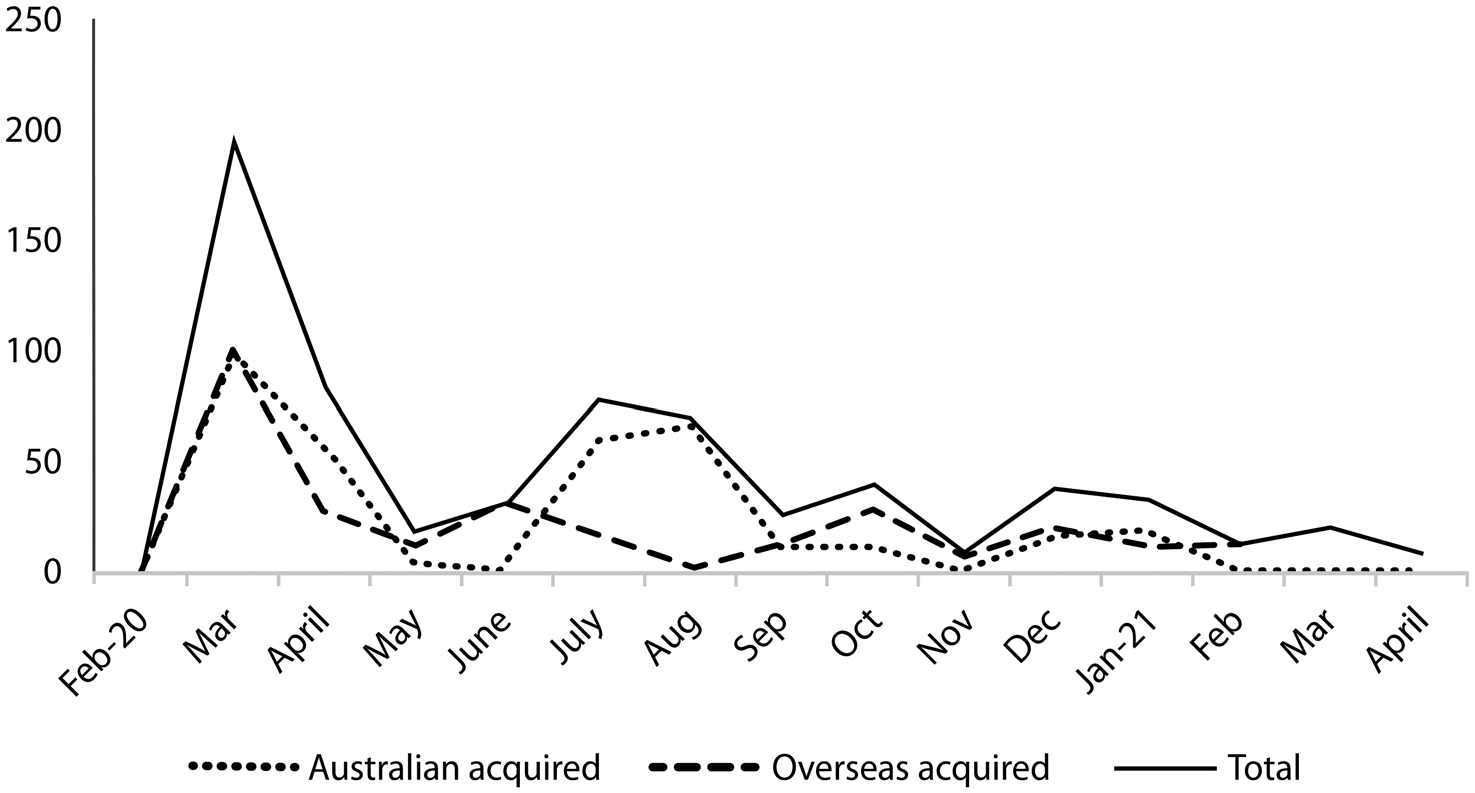
COVID-19 cases in Western Sydney Local Health District February 2020 – April 2021. Study participants interviews from 22nd October 2020 to 15th April 2021. (Source https://data.nsw.gov.au/data/dataset/covid-19-cases-by-location)

Participants were recruited at a tertiary referral hospital in WSLHD, NSW, Australia. WSLHD has a high migrant population with 58% of women who use the maternity service born in non-English speaking countries [16]. The recruitment hospital has approximately 5000 births per year [16]. During the study period there was a low prevalence of COVID-19 in the community with a total of 149 cases in the health district including two women recorded to have been COVID-19 positive during pregnancy (Fig. 1) [4]. Community ‘lockdown’ orders were in place for the state of NSW for seven weeks from 30th March 2020. However, hospital COVID-19 mitigation restrictions remained for the study period including hospital entrance health screening, patient only waiting rooms, no support people in ultrasounds and only one support person permitted in the birth unit.

### Participants

**Fig. 2:**
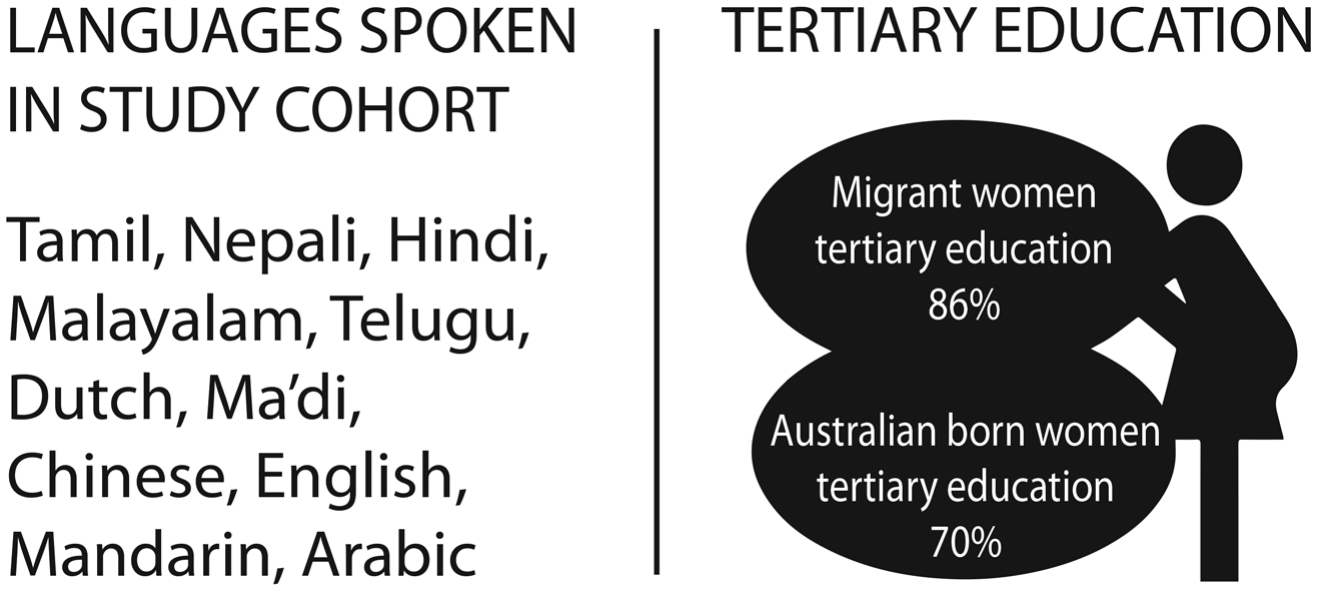
Characteristics of the 24 study participants

There were 24 participants interviewed, with 22 interviews conducted antenatally and 18 postnatally. Antenatal inclusion criterion was gestation greater than 34 weeks and postnatal inclusion required that the baby was not a hospital inpatient. Maximum variation purposive sampling was used to ensure a culturally diverse participant voice was represented that reflected the local population (Fig. 2) [17].

### Data collection

Three sources were used for data collection: interviews, study specific questionnaires (Additional file 1) and routinely collected maternity data. The survey instrument was developed by the investigators and edited after pilot testing for readability with eight culturally diverse women. Cultural groupings were based on Mothers and Babies annual report New South Wales with known local demographic considerations [16].

Antenatal and postnatal interviews occurred over a six-month period from October 2020 to April 2021. All participants were offered an option of face to face (when COVID-19 restrictions allowed), telephone or video interview at times convenient for them. Of the twenty-two antenatal interviews eleven were face to face, six were video interviews and five interviews were completed by telephone. Eighteen postnatal interviews were conducted with participants, 17 by telephone and one by video. Recruitment ceased when sufficient rich data was collected from a diverse range of participants to provide insight into the research question. The interview times ranged from 30 to 75 min antenatally and 15 to 70 min postnatally interviews. All interviews were transcribed by a professional transcription service. Interviews were conducted by the research midwife first author (SJM) with progress and oversight provided by an expert qualitative researcher (JM).

### Data analysis

Quantitative data was collated from surveys and presented as descriptive statistics. Data from transcribed interviews were coded and analysed thematically using an inductive approach. Analysis was conducted in six non-sequential phases as described by Braun and Clarke [18]. Deep familiarisation with the data by the first author was the initial phase that included reading and rereading all interview transcripts, discussing interviews with the senior author (JM), reviewing field notes, reviewing transcription with audio to correct transcription errors. Iterative phases for data analysis were: generating codes; exploring initial categories and themes; reviewing initial themes; finalising and naming themes, and producing the report [19]. Two authors (SJM, JM) agreed upon and defined codes (derived from both explicit and implicit meanings), categories and themes. Themes were reviewed and consensus reached by a third author (NG) acting as a content expert as the maternity cultural liaison officer in the study hospital (MLO). NVivo (release 1.5.1) was used for data management and assistance with analysis.

Personal, research and clinical experience of investigators was taken into reflexive consideration and trustworthiness during all phases of the research process. All investigators had disruption to their professional and personal lives through the lived experience of the pandemic and this was also reflected upon by investigators throughout the project design, data collection, analysis and interpretation phases.

## Results

The study covers a low COVID-19 prevalence period in the local health district (Fig. 1). Antenatal interviews were conducted with women between 34 and 39.4 weeks gestation and postnatal interviews 5–12 weeks after birth (Table 1). There were 14 overseas born participants and 10 Australian born. One of the overseas born participants stated she was a refugee. Five out of the 10 Australian born women stated they had partners who were migrants. Most participants were tertiary educated (19 out of 24, 79%, Fig. 2). Most Australian born (7 out of 10) and migrant (8 out of 14) women were employed fulltime, part-time/casual or were on maternity leave.

**Table 1:** Antenatal and postnatal interview and participants characteristics during the low prevalence first year of the COVID-19 pandemic in Western Sydney, Australia

Participant	Country of birth	Self-identified cultural group	Primiparous	Years lived in Australia		Weeks of gestation antenatal interview	Weeks postpartum at interview
P1_OS	India	SA	Yes		4	.	8
P2_OS	Nepal	SA	Yes		2	37.1	9
P3_OS	Nepal	SA	Yes		3	39.1	10
P4_OS	India	SA	Yes		2	38.5	12
P5_OS	India	SA	Yes		4	36.5	9
P6_OS	India	SA	Yes		3	.	10
P7_OS	India	SA	Yes		2	38	9
P8_OS	Nigeria	African	No		5	34	.
P9_OS	Netherlands	Dutch/Irish	No		6	36.6	7
P10_OS	Philippines	SEA	No		10	39	10
P11_OS	South Sudan	African	No		10	35.1	8
P12_OS	China	NEA	No		5	36.6	7
P13_OS	India	SA	Yes		10 months	39.1	.
P14_OS	China	NEA	No		8	35.4	12
P15_AustB	Australia	ES	No		.	36.6	
P16_AustB	Australia	ES	No		.	37	10
P17_AustB	Australia	ES	No		.	36.2	.
P18_AustB	Australia	ES	No		.	34.3	6
P19_AustB	Australia	ME/ES	Yes		.	34.6	9
P20_AustB	Australia	ES	No		.	34.3	7
P21_AustB	Australia	ES	Yes		.	39	.
P22_AustB	Australia	ES	Yes		.	39.4	6
P23_AustB	Australia	ES	Yes		.	36.6	5
P24_AustB	Australia	ES	No		.	38.6	.

OS = Overseas born AustB = Australian born SA = South Asian SEA = Southeast Asian NEA = Northeast Asian.

ES = English Speaking: Australia, United Kingdom. New Zealand or America.

ME/ES = Middle Eastern/English Speaking.

### Themes

Among this well-educated cohort, we identified four major themes when exploring support for women in a high migrant population during the first year of the COVID-19 pandemic. These themes were: significant disruption and loss of peripartum support during the COVID-19 pandemic that had ongoing impact for migrant women; holding on by a virtual thread; supportive employers making a difference, and impact on mental health of both participants and partners. Other themes identified were: husbands/partners filling the support gap; building family bonds; a lucky few with neighbourhood support close and plentiful; COVID-19 fear and baby safety.

### Significant disruption and loss of peripartum support during the COVID-19 pandemic that had ongoing impact for migrant women

No migrant women in the study had access to their mother’s physical tangible support due to COVID-19 related travel restrictions. Half the migrant women in the study described feeling unsupported or having less support than they would have wanted. This was the case in the antenatal interviews and remained so for the postnatal interviews when COVID-19 restrictions had largely been removed in the community; “…life has somehow stopped, and I really want my mum to visit me as soon as possible” (P4 India Postpartum). Half the Australian born women also described a lack of support during pregnancy when hospital, local and interstate COVID-19 related travel restrictions were present. However, satisfaction with support improved for Australian born women postnatally when these restrictions were eased.

*So now that all the COVID restrictions, especially with the amount of people in your households and everything like that, have been completely reduced, we’ve had more support than we can handle*. (P24 Australian born (Aust) Postpartum)

Issues with access to support continued for most migrant women into the postnatal period with no difference between multiparous or first-time mothers. Missing out on overseas family support appeared to be similar for women who were new migrants and for those who had migrated more than five years previously. The survey identified most (64%) migrant participants had planned on receiving supportive visits from overseas relatives. The duration of planned visits was to have been from three to six months.

*But this time, due to COVID, no one is able to [come]. [Last baby]…we have four parents here and then this year no one is able to come...Yeah, most worry is I don’t have any family support, they restrict me from getting supports from my family.* (P14 China Antenatal)

First time migrant mothers stated they missed instructional support and multiparous women expressed missing emotional support and child-care support for older children. Two migrant women stated they paid for in-home help to fill the gap left by absent relatives. One had assistance for a few hours a day, the other had live-in help for six weeks. The participant with in-home help felt she had not missed her mother as much as anticipated in the antenatal period as the carer had provided valued professional guidance for newborn care. However, she also said paying for this help had been a significant economic burden on the family.

Almost all migrant women when interviewed postpartum felt they missed their mother but by using various strategies were able to cope better than they had thought they would during the antenatal period.

*So, yeah, it turned out really good. Day by day started falling in place, we got sort of prepared that, no, our in-laws couldn’t come, no one from the family is going to come, so we have to be by ourselves, borders are not going to open. We were very well prepared, we were by ourselves, we have to do things on our own, and by God’s grace we have really good friends around who stepped forward for our support.* (P6 India Postpartum)

### Husbands/partners filling the support gap; “He did a fantastic job. I’m so blessed. I’m totally blessed”

Most (13 out of 14) migrant participants stated their partners were integral to them feeling supported during their pregnancy and when caring for their newborn; “I really don’t have much support, … only my husband he is there for me” (P11 South Sudan Antenatal). In contrast only a minority (3 out of 10) of Australian born women discussed partner support. Migrant women described that their partners had stepped in to provide support to compensate for the loss of traditional support mothers and other relatives would have provided.

*…it was my husband who stepped into my mother’s role, or a true companion role, I could say, that he started cooking, he started learning how to cook and take care of me. He did a fantastic job. I’m so blessed. I’m totally blessed.* (P6 India Postnatal)

There were other factors during the pandemic that facilitated partner support such as opportunities created from husbands/partners working at home. More than half the participants described the supportive benefits of their partners’ capacity to work from home.

*Just my husband is home. [Laughs] So easy. He’s always available. If it was not COVID, then my mom would have been here. So that would have been a different story. But my husband is home, working from home, so he’s available 24/7. So, if I find any difficulty or trouble, anything really, to my mood swing or whatever, I can go to him.* (P4 India Antenatal)

### Building family bonds - the benefit of missing support; “*…it made us more self-dependent”*

#### Gender equity and opportunity

Women described that their partners had benefited from the extra family time that may not have occurred without COVID-19. There were increased opportunities for husbands to not only understand the needs of their partners during the peripartum period and provide support for them, but also engage in care for their newborn and older children. One study participant described that the pandemic had provided a unique opportunity for them to understand gender roles and for her husband to be a greater part of the pregnancy journey.

*[My husband]...started to understand more like, OK, what exactly a woman is, what exactly a man is. Those things are something I believe it’s really hard… And he actually know what an importance a woman is, what a pregnancy is, what a baby delivery is. So, I think that makes your relationship and your bonding more stronger.* (P6 Postnatal India)

A participant explained during the postpartum interview she had found some benefits to relatives being unable to visit including not having to deal with the stress of negotiating inter-family harmony; “I don’t have to worry about the relationship between in-laws or my parents and me because sometimes we have different opinions [on baby care] [laughs]” (P14 China). She further explained the support from her husband with caring for her first-born child had strengthened their relationship and their family unit. Another participant explained COVID-19 travel restrictions benefits included staying with her husband rather than separating for the birth.

*I would go to India for the delivery, and come back after some time, my in-laws or my parents would have been here, and he would have gone to work at the time…Yeah, he would have missed everything, yeah.* (P1 India Postpartum)

Illustrating the support from her husband to care for both her needs and the newborns’, one participant described the extent of organised leave he had taken to provide the practical support that would have otherwise been given by relatives; “…my husband has taken off two months, and after two months definitely I have to look after me, like for myself and as well as for the kid” (P3 India Antenatal). The practical skills gained of infant care for both parents that would have been relegated to others were also appreciated by some participants. In antenatal interviews, the forced parenting independence and infant care skill acquisition was the cause of some anxiety, that participants’ parents would not be present to be ‘taking charge’, but this was ultimately valued postnatally.

*…it’s a positive, but then you’re learning the hard way…when someone is there taking charge, they are thinking, it’s not you who are thinking, you’re just following the lead. So you are not thinking and you’re not, you know, trying to understand what’s happening, but now you have to think and you have to get things done.* (P5 India Postpartum)

Families that had previous experience of live-in help from relatives were aware of missing that support but also understood the opportunities of a different experience with partners, for example having the time and space to be more engaged with infant care.

*And he’s very excited as well because the last one obviously we had all the family and he didn’t necessarily have to step up …there was times where he felt like he – my mum would take over or something like that. Well, he’s quite excited about the fact that he will have to do night feeds and he will have to change all the nappies.* (P9 Netherland Antenatal)

### Holding on by a virtual thread; “*…thank God for technology”*

Almost all participants described a high level of virtual contact with families, particularly their mother. For both Australian born and migrant women, contact with their extended family was daily for approximately two thirds of women in the study (16 out of 24), with three women describing virtual contact as being several times a day. More migrant women had daily contact than Australian born women. All other participants stated contact with family was at least weekly. Virtual contact was through the use of audio only telephone, text or video (Table 2). Participants’ definition of ‘frequent’ speaking or contact varied from ‘multiple times a day’ to weekly and the length of contact ranged from five minutes to several hours.

*…she was pretty excited to come here and help me through, but unfortunately it could not happen. But I speak to her every day. I do a video call and then we speak for hours.* (P4 India Antenatal)

**Table 2:** Quantitative Survey data participant peripartum support details during the low prevalence first year of the COVID-19 pandemic in Western Sydney, Australia

Participant	Planned length of stay for live in help (months)	Frequency	Contacts overseas relatives
P1_OS	6	Daily	Facetime
P2_OS	6	Every few days	Viber
P3_OS	.	Every few days	Viber
P4_OS	3	Daily	N/A
P5_OS	6	Daily	WhatsApp
P6_OS	.	Daily	WhatsApp
P7_OS	3	Daily	WhatsApp
P8_OS	.	Every few days	Voice phone
P9_OS	1	Every few days	WhatsApp
P10_OS	.	Occasionally	Facebook Messenger
P11_OS	12	Every few days	Voice phone
P12_OS	.	Every few days	N/A
P13_OS	.	Daily	N/A
P14_OS	6	Every few days	WeChat
P15_AustB	.	.	.
P16_AustB	.	.	.
P17_AustB	.	Weekly	Facetime
P18_AustB	.	.	.
P19_AustB	.	Daily	WhatsApp
P20_AustB	3	Every few days	Voice phone
P21_AustB	1 week	.	.
P22_AustB	.	.	.
P23_AustB	.	.	.
P24_AustB	.	.	.

OS = Overseas born AustB = Australian born N/A = not available

One Australian born participant described the routine daily video contact with her mother who lives interstate: “She likes to have breakfast with us… She’ll have her coffee ready and then she watches my son eat his breakfast and make a mess” (P20 Aust Antenatal).

Distance did not appear to be associated with the frequency of virtual family contact for both Australian born or migrant women. One Australian born woman who had her mother, relatives and friends in close proximity, had virtual contact by telephone with her mother 2–3 times per day, her sister multiple times per day and her brother, who lived interstate, weekly. A study participant described daily contact with various family members who had migrated to other countries: “All family in different four countries, every day someone is checking” (P6 India Postpartum). Three participants spoke of frequent video calls that fostered a virtual relationship with grandchildren and assisted with virtual childminding.

…*they want to see her and talk to her. So, we make a video call, and we fix in a stand, and make them to speak with her. So she babbles again to them. So they feel little relieved, and they feel little happy… I’ll do my cooking things, and I’ll do all the housework things. And so that they could interact with her, and they make her distracted…* (P1 India Postpartum)

Participants stated frequent inexpensive virtual contact with their support networks was also beneficial to their mental health, particularly during COVID-19 lockdown periods with reduced opportunities to socialise: “I was in (virtual) contact with friends and family frequently, I didn’t feel much loneliness or any sort of things during COVID (lockdown)” (P13 India Antenatal).

However, some women expressed the frequent virtual contact did not replace the desire for physical presence. One participant discussed the loss of child-minding support; “they can just only tell us what to do but they cannot help…” (P14 China Postpartum). Most women from overseas also sought virtual guidance to maintain cultural connection and adherence to traditions.

The content of conversations with relatives for the majority of Australian born women differed from overseas born women. Australian born participants stated the content was primarily connection and emotional support rather than cultural connection and adherence to traditions; “…sometimes we’ll just sit there and share recipes of what we’re making for the week, just to kind of have that communication” (P23 Aust Antenatal).

Not all migrant women had access to reliable frequent virtual support due to both financial constraints and overseas relatives having limited or no access to technology, which reduced capacity for frequent virtual support and contact. The difficulty for some migrant women to connect to family members in low-income countries was discussed. One study participant from South Sudan described it was easy and free to use phone apps to contact her sister but connecting to her mother was problematic; “My mum is in a village, and I have to put a credit and call and sometimes I do not have credit”.

### Two-way virtual support

The desire for, and disruption of, frequent support was two-way. Both Australian born and migrant participants stated that as well as receiving support, they were also having to provide support virtually that they would have provided in-person without COVID-19 restrictions. For example, one Australian born woman spoke of providing virtual support for her mother:

*Mum has leukaemia, so I can’t see her during COVID… I think it’s just one of those two-way things where we just feel so horrible about the whole situation, and that we can’t – we aren’t doing enough for each other.* (P23 Aust Antenatal)

Virtual contact was the only option due to travel restrictions and the vulnerability of her unwell mother. However, she was able to facetime daily from ten minutes to two hours; “I mean half the time we just lay there, and I wait for the baby to move so she can see it” (P23 Aust Antenatal). One migrant woman described her father’s illness and then his death. She was unable to be present to support her mother in the Philippines but could at least provide some support virtually.

*…you know, thank God for technology. We can still talk to them and call them, and Mum gave us updates when Dad was in hospital, in the ICU, and all those things. So we were still there but we were not there, if that makes sense.* (P10 Philippines Antenatal)

### Supportive employers making a difference; “…the work that he’s with now has been amazing”

There were eleven women who specifically discussed support they or their partner received from employers. All participants who discussed paid employment stated during the COVID-19 pandemic they felt supported in their pregnancy by their workplace; “Even my manager told me like, ‘If you need to take more breaks, take it.’” (P5 India Antenatal). No participant reported that their management or their husband’s workplace had been obstructive with requests concerning pregnancy care or leave.

Working from home for most participants was valued and viewed positively. Women discussed feeling more relaxed as they no longer had to spend time on crowded public transport with the associated COVID-19 risk. Women stated they benefited financially from reduced expenditure on transport and clothing.

### The neighbourhood village: a lucky few with support close and plentiful; “…*we’ve had more support than we can handle.”*

Almost all Australian born women who had relatives living nearby felt well supported. Migrant families drew support from local friends, siblings or their own or partners’ families but still did not feel as supported as Australian born women. One participant had both her family and her husband’s family living close and felt COVID-19 had not changed anything for her. At both the antenatal and postnatal interview she felt well supported by her close family; “Yeah, it’s been great [support]. I’ve got a great family; got a very large family, and my partner’s family is great as well… I just live right around the corner from my parents, so we were over there quite a lot” (P16 Aust Antenatal).

### Impact on mental health for both parents; “…*It was very lonely”*

Both migrant and Australian born women discussed the impact the pandemic and restrictions had on support and their mental health. Loss of joy around the pregnancy experience was discussed: “It’s been very different. Otherwise I would have really enjoyed it” (P4 India Antenatal). The emotional burden of living in the pandemic was difficult for many women; “…it was all just kind of one thing after another after another, where it’d been this extreme emotional rollercoaster for myself and my husband” (P23 Aust Antenatal). The direct impact of hospital restrictions on partners attending some appointments and ultrasound impacted the mental health of the family and created uncertainty for women’s access to support; “Like he ended up in tears when we couldn’t see the first ultrasound [together], and he wasn’t allowed in” (P23 Aust Antenatal).

Three women had a history of anxiety or depression which was noted on routinely collected data at hospital booking. Two of these women stated they felt well supported postnatally and antenatally on interview. No women in the study cohort scored > 10 on the Edinburgh Depression Score (EPDS) at booking-in visit with the midwife. Two participants on interview disclosed they had professional help for mental health concerns, neither disclosed a history of mental health issues at routine booking visit or scored > 4 on the EPDS. One of these participants was diagnosed with depression for the first time during the antenatal period and stated the loss of her husband’s presence and support during appointments contributed to her depression and feelings of isolation.

*Just with the COVID restrictions the experience wasn’t great. Just mentally, I think, more than anything, COVID impacted it. I had a really healthy pregnancy, I didn’t really have any issues, but mentally it was a bit of a challenge… just a huge mental drain.* (P23 Aust Antenatal)

During the study period, only patients were allowed in the antenatal waiting room. One participant explained these hospital restrictions had left her partner feeling like a criminal; “He always uses that phrase: ‘I feel treated like a criminal’ ” (P10 Philippines Antenatal). The threat and uncertainty around the potential loss of partner support was a source of concern and stress for women; “I was worried that he wouldn’t be able to be there or that I would have to go through labour on my own” (P9 Netherlands Postpartum).

### COVID-19 fear and baby safety above all else: “…so worried, that if I get COVID, it would kill the baby”

Overall, the majority of study participants (18 out of 24) stated in antenatal interviews that their main concern regarding COVID-19 was the safety of their baby while pregnant or as a newborn: “…what if I get it, it’s going to pass onto my baby. And I’m not scared for myself” (P6 India Antenatal). This concern for their unborn baby restricted most participants’ movements outside the house beyond the lockdown and lasted throughout their pregnancy. This was the case for most migrant and Australian born women; “I am very scared to go out and risk meeting other people…We rarely went out’ (P10 Philippines Antenatal); ‘I was so, so worried, that if I get COVID, it would kill the baby” (P23 Aust Antenatal).

The strong public health messaging in the community around the importance of hand sanitising and wearing face masks left four women feeling unsupported with lack of information on how to keep their baby and children safe from COVID-19.

*We cannot wear mask or sanitise the hands of the baby. We can do it but we cannot do it for the baby… What are we going to do with the baby, like how are we going to ensure the safety of the baby is a question and concern.* (P13 India Antenatal)

Many participants spoke in the antenatal interviews of ways to mitigate infection risk. Three women discussed the fear of the effect COVID-19 infection may have on their unborn child which resulted in themselves or their husband changing or stopping work which could have a significant financial impact:

*When I have my pregnancy last time I was working, but this time when I found I was pregnant I quit my job because of the COVID-19 just to keep safe… I would like to work because I can earn money for the family, but yeah, I have to balance the safety.* (P12 China Antenatal)

Most migrant women postpartum discussed their primary concern with COVID-19 had shifted away from the risk of COVID-19 infection to the shut borders and missing support: “…the major thing is my family. I’m, like, I’m worried my baby can’t see my family” (P2 Nepal Postpartum).

## Discussion

This study identified differences between migrant and non-migrant women in their experience of peripartum social support during the COVID-19 pandemic. We found that migrant women were significantly impacted during the pandemic both antenatally and postnatally, primarily due to the loss of support from disrupted international travel. Migrant populations have been disproportionately impacted by the COVID-19 pandemic with studies providing evidence of increased burden of COVID-19 disease, mental health and socio-economic consequences in both high and low-income countries [1, 19–23]. It is essential to understand women’s experience during the pandemic to assist in providing optimal support, planning future pandemic risk mitigation and identifying potential factors that affect obstetric and other health outcomes.

Supportive social networks are important as a buffer for stressful life events and have been shown to improve cardiometabolic health outcomes [10, 24–26]. Optimal social supports are mitigators for the known impact that stress, disasters and pandemics can have on long term cardiometabolic health as well as a wide range of immediate benefits, including mental health and child development [27–34]. Disrupted support during the pandemic may also influence perinatal outcomes [35]. Healthcare systems understanding, facilitating, and supporting these buffers for long term health, may prove increasingly important.

This study identified a disrupted support network for participants but also identified how technology played an integral part assisting women to remain connected to family. Virtual support has been identified as a mediator to improved perinatal mental health and social support both before and during the pandemic [33, 36, 37]. We found virtual support acted as a significant buffer to stress providing emotional and informational support, as well as improving mental health. Clinicians should continue to find avenues to encourage and facilitate the use of virtual social support during the peripartum.

The cultural expectation of many migrant women is their mothers or relatives will provide extensive support postpartum to facilitate resting and care of the newborn. For some cultural groups this may involve 30 days of no cooking or domestic cleaning duties for new mothers, with the expectation of complete physical rest and a focus on caring for the new baby [14, 38]. This study identified migrant fathers were facilitating new mothers’ rest and this assisted with maintaining valued cultural practices and connection to countries of birth.

Our research has highlighted the impact of missing relatives within culturally driven gendered domestic roles, now filled with ‘husbands/partners stepping up’. This study has illustrated that opportunities to work from home facilitated filling of a domestic labour void by partners. Women described that COVID-19 delivered unexpected gains of opportunities for men to participate meaningfully in partnering more equally in parenting and domestic life, with research suggesting health benefits for both [39].

We have identified most women during the peripartum period had contact with their mother or close relatives frequently and are highly reliant on them for social support. We found a generation that has access to and utilised instant communication facilitating support. The shift to communicating virtually during the pandemic was embraced by our families. However, other research has shown the pandemic drove maternity care away from patient centred care and focused on preservation and safety of staff without providing meaningful alternative options to support and connect clinicians with women and partners during the pregnancy journey [9, 40, 41]. Understanding and leveraging existing virtual patient communication preferences in clinical care may strengthen care provided. Virtual emotional and informational support has been identified in this study to improve mental health and be highly valued by all women, but particularly women in our migrant population. Potentially utilising and leveraging this connection for family group counselling intervention programs and education may improve health outcomes as well as engagement with maternity services. Exploring new ways to support women using eHealth mental health programs may also offer some feasible and affordable ways to improve the health impact of isolation and depression during the peripartum period, particularly for migrant women [42, 43].

We found the adverse impact on mental health for migrant women was largely driven by the loss of valued overseas tangible support. Other research has identified that during the pandemic higher levels of social support were protective for adverse perinatal mental health [44, 45]. Migrant women are potentially more at risk due to barriers to mental health diagnosis and treatment [46, 47]. Antenatal screening has been recommended to identify women who have low levels of social support and are more vulnerable to mental health disorders, but this has been problematic both in the development of care pathways and an appropriate universal screening tool [48, 49]. We identified women with a significant mental health concern requiring treatment in our cohort who were not identified at early pregnancy screening. Clinicians need to reassess patients’ mental health status throughout the perinatal period and offer appropriate clinical support. Pandemic preparedness should include development and use of social support screening and care pathways particularly for vulnerable and migrant women.

Similar to other studies, we identified that hospital restrictions on support people and women fearing the impact of COVID-19 on their unborn babies also negatively influenced women [9, 50]. Some women chose loneliness to protect their baby from potential COVID-19 infection. The increased isolation during pregnancy beyond lockdown periods identified in this study may provide some evidence for potential drivers for the reduction in spontaneous preterm births found in our district [15]. It has been hypothesised COVID-19 isolation may reduce exposure to pathogens associated with preterm birth [15, 51].

### Strengths and limitations

A strength of this study is utilising an interview process including both antenatal and postnatal interviews with the same participant to clarify and compare pre- and postnatal experience. Limitations include the cohort was highly educated potentially with greater capacity for partners to be working from home and have the financial means for access to virtual support. Another limitation is the follow-up interviews were only up to 12 weeks postpartum. Migrant women’s experience of partner support may well be different at one year postpartum when partners are no longer on parental leave or unable to work from home.

## Conclusion

During the pandemic there has been a growing body of research highlighting the disparities in health outcomes for migrant populations and this study provides further evidence of the vulnerabilities of migrant women. Half the migrant women in this study felt unsupported or reported less support than they would have wanted due to pandemic restrictions. We found no difference between migrant women who felt unsupported postpartum if they were multiparous, primiparous, new migrants or had migrated greater than five years ago. In contrast to migrant women, perceptions of support improved postpartum for Australian born women. These findings demonstrate the wide-spread devastating impact the pandemic has had on our migrant families and highlights the need for perinatal support assessment in pandemic preparedness. However there have been some benefits including gains in gender equity and therefore the need for care providers to be mindful of gender role assumptions in migrant families. Assessing ongoing disruption to support experienced by migrant women will assist with clinical care, as well as planning for future pandemics and the potential impact on current and long-term health associated with impaired support.

## Electronic supplementary material

Below is the link to the electronic supplementary material.

**Additional file** 1

## Data Availability

Due to privacy issues of participants individual dataset is not publicly available. Summary data is available on reasonable request from the corresponding author if compliant with local ethical guidelines.
