# Supplementary material for: Women’s experience of perinatal support in a high migrant Australian population during the COVID-19 pandemic: a mixed methods study
Source: BMC Pregnancy Childbirth. 2023 Jun 9;23:429. doi: 10.1186/s12884-023-05745-9 (PMC10250860; doi:10.1186/s12884-023-05745-9)
Supplement: Supplementary file 1 — Additional file 1 [file 12884_2023_5745_MOESM1_ESM.pdf]

Additional file 1

Women's experience of perinatal support in a high migrant Australian population during the COVID-19 pandemic: a mixed methods study

Survey used for study participants

STUDY SURVEY

Exploring Perinatal Care and Cultural perspectives during the COVID-19 pandemic

Please complete the below questions (tick boxes)

Country of Birth :__________________<br><br>Language spoken at home :__________________<br><br>Years lived in Australia :__________________	**Cultural group**<br>□ **English Speaking** Australia, UK, New Zealand, America<br><br>□ **Other** Please list cultural group: ________________________	□ **South Asian** India, Sri Lanka, Pakistan, Afghanistan, Bangladesh, Bhutan, Maldives, Nepal	□ **North East Asian** China, Japan, Taiwan, Mongolia, North Korea and South Korea
**Highest level of education**<br><br>□ No formal education	□ High school or lower<br><br>□ Trade certificate/ Certificate III	□ Diploma or degree (Bachelor's, Honours)<br><br>□ Other_______	□ Postgraduate (Master's, PhD)
**Paid Employment**<br>□ Fulltime	□ Part-time	□ Casual	□ Looking for employment
Number of hours in paid employment per week ___ Hrs	□ Temporarily stood down due to COVID-19	□ Stood down due to COVID-19	□ On maternity leave
Has your income been affected by COVID 19?	□ Yes<br>□ No	Comment:	
If you have a husband/ partner are they employed?	□ Fulltime<br><br>□ Temporarily stood down due to COVID-19	□ Part-time<br><br>□ Stood down due to COVID-19	□ Casual
Has your partner/husband's income been affected by COVID 19?	□ Yes<br>□ No	Comment:	
Are you or have you been a refugee?	□ Yes<br>□ No	Comment:	

Present relationship?	□ Living with father of baby<br>□ Separated/Divorced not living with father of baby		
What other people do you live with?	□ Mother □ Friend □ Relative<br>□ Mother-in-law □ Other____________		
Did you plan to have someone other than your husband live with you to help you with the baby?	□ Yes<br>□ No	***If answered Yes***<br><br>This person was to be:<br><br>How many months did they plan to stay?<br><br>Did they plan to come from another country to stay with you?<br><br>Did COVID 19 stop them coming?	□ Mother<br>□ Mother-in-law<br>□ Friend<br>□ Relative<br><br>______ months<br><br>□ Yes<br>□ No<br><br>□ Yes<br>□ No
Do you access the internet from home?	□ Yes<br>□ No	Include internet access using desktop/laptop computers, mobile or smart phones, tablets, music or video players, gaming consoles, smart TVs etc	
Do you speak to relatives overseas?	□ Yes<br>□ No<br><br>*If yes*	Please circle how you speak to them: voice phone, Skype, Zoom, face-time, Wechat, Other________<br><br>How often?	□ Daily<br>□ Every few days<br>□ Occasionally<br>□ Almost never
For pregnancy care appointments during COVID pandemic would you like to have the option of:	□ Telephone appointments<br><br>□ Video (webcam) appointments	□ I only like face to face appointments	

Thank you for completing this survey prior to your interview with the researcher
